# Novel Ground-Up 3D Multicellular Simulators for Synthetic Biology CAD Integrating Stochastic Gillespie Simulations Benchmarked with Topologically Variable SBML Models

**DOI:** 10.3390/genes14010154

**Published:** 2023-01-06

**Authors:** Richard Oliver Matzko, Laurentiu Mierla, Savas Konur

**Affiliations:** Department of Computer Science, University of Bradford, Bradford BD7 1DP, UK

**Keywords:** synthetic biology, systems biology, CAD, multicellular simulation, stochastic Gillespie, unreal engine, chemical reaction networks, automation, biophysics, SBML

## Abstract

The elevation of Synthetic Biology from single cells to multicellular simulations would be a significant scale-up. The spatiotemporal behavior of cellular populations has the potential to be prototyped in silico for computer assisted design through ergonomic interfaces. Such a platform would have great practical potential across medicine, industry, research, education and accessible archiving in bioinformatics. Existing Synthetic Biology CAD systems are considered limited regarding population level behavior, and this work explored the in silico challenges posed from biological and computational perspectives. Retaining the connection to Synthetic Biology CAD, an extension of the Infobiotics Workbench Suite was considered, with potential for the integration of genetic regulatory models and/or chemical reaction networks through Next Generation Stochastic Simulator (NGSS) Gillespie algorithms. These were executed using SBML models generated by in-house SBML-Constructor over numerous topologies and benchmarked in association with multicellular simulation layers. Regarding multicellularity, two ground-up multicellular solutions were developed, including the use of Unreal Engine 4 contrasted with CPU multithreading and Blender visualization, resulting in a comparison of real-time versus batch-processed simulations. In conclusion, high-performance computing and client–server architectures could be considered for future works, along with the inclusion of numerous biologically and physically informed features, whilst still pursuing ergonomic solutions.

## 1. Introduction

The objective of Synthetic Biology has been described as the utilization of biology technologically [1], especially from the DNA level, for essentially unlimited possible outcomes. The challenge explored here is in elevating CAD (computer assisted design) to the multicellular level. Data is available within various repositories upon which models and simulations can be constructed. In fact, bioregulatory models acquired from repositories can be harnessed and applied dynamically to spatiotemporal simulations [2]. Thus, computers are poised for the computer assisted design of blueprints, upon which in silico proofing can be performed, with potential parameter optimization [3] and finally laboratory application and/or verification [4] from ‘in silico first’ efforts.

The integration of the NGSS (Next Generation Stochastic Simulator) [5] into two unique multicellular simulation layers was pursued, with subsequent benchmarking of performances with and without this subcellular processing layer. In this way the scalability, tractability and feature differences could be assessed, commencing at the personal computing level. With NGSS integrated into the Infobiotics Workbench (IBW) platform [6], the present work pursued the extension of this Synthetic Biology CAD system to the multicellular context through the mutual SBML [7] model exchange format. Multicellularity was found to be absent or limited in such suites [1,6,8], especially with regards to compelling physical, spatiotemporal 3D solutions. Coupled with a phenomenological assessment of cellular behavior and the respective microenvironment (Section 1.3), the work sets the foundation for promising developments towards increasingly realistic, instructive and useful multicellular simulations with diverse, emergent spatiotemporal potential. Such elucidation coordinates with the phenomenological approach that was encouraged in the literature [9].

Unreal Engine 4 (UE4) as a real-time platform (including physics) for multicellular simulation and CAD design was assessed as contrasted to a rules-based batch-processed dynamic mesh generation approach. The distinction between real-time and batch processed performances in multicellular simulation are not clear from the literature and is one of the critical design aspects to consider when developing a multicellular simulator (see Section 1.4). The work would demonstrate the call for batch processing over real time solutions, as well as performance profiles of parallel NGSS processing that highlighted the need for future high-performance computing (HPC) implementations for subcellular models (see Section 3.3) in the pursuit of expanding IBW.

The features of multicellular simulation as well as the data exchange technologies discussed in this section were considered for the construction of the multicellular layers presented in the methods section (Section 2) as well as for the subcellular bio-chemical processing layer used in association with IBW’s stochastic simulator (NGSS).

Therefore, this work pursued the extension of Synthetic Biology CAD towards multicellularity, comparing different methodologies and approaches, including the use of UE4 for modern graphics and design, novel use of NGSS, furthermore reviewing computational, physical and biological features and integrating them for user manipulation via a “Cell Editor”, whilst also ensuring 3D capabilities, ease-of-use, ergonomic GUIs and native Windows accessibility unlike other agent-based platforms [10,11]. Additionally, such platforms tended to emphasize specific physical dynamics, whilst this work emphasized SBML biochemical model integration, phenotypic modulation and benchmarking of chemical reaction networks (CRNs) in association with the originally designed multicellular simulation layers, also with automated CRN generation of varying topologies. Client–server architecture would also present as a solution to a variety of challenges, including ergonomics and performance, as explored in parts of this work. Also discussed are other intricacies, such as design features, that should prove useful to developers.

This research article for Genes is an expansion of a selected conference paper [12].

### 1.1. Bioregulatory/Metabolic Simulations and Exchange Standards

SBML is an exchange format designed to exchange modeled data within Systems Biology and between computational modeling and metabolic simulation tools [7]. There are a number of simulators available for the chemical level that can solve various biological computations, such as biochemical reactions or state transitions (e.g., transport). These simulators tend to be designed for solving SBML models, which can capture the mathematics of biochemical reactions and regulatory models, and such simulators have been used to compute subcellular models within multiscale, multicellular simulations [13]. They can utilize various biochemical simulation modalities [14], including ordinary differential equations (ODEs), stochastic algorithms and flux balance analysis, and may even possess parameter estimation capabilities. Whilst stochastic approaches are computationally expensive, they are considered more principled than ODEs, and the justification for their use has been given [15] by the argument that stochastic models can capture the noise of biochemical systems, whilst also effectively fulfilling the modelling of genetic switches. It was argued that deterministic ordinary differential equations are incapable of fulfilling these objectives effectively. Hybrid approaches have also been pursued [14], where low particle numbers suited stochastic simulations whilst faster reactions containing more reactants could be solved deterministically. Boolean models provide a convenient alternative solution by avoiding the need for kinetics data [16].

### 1.2. Gillespie Stochastic Simulation Algorithms and Performance Prediction via SSAPredict

NGSS [15] possesses one approximate and 8 exact Gillespie stochastic algorithms [6] and was notably incorporated into the Infobiotics Workshop Synthetic Biology suite. Stochastic Simulation Algorithms (SSAs) behave equivalently to Chemical Master Equations; a set of probabilistic differential equations. NGSS can operate on a single logical core (i.e., serially) or on multiple CPU logical cores, and outputs average concentrations over one or more parallel runs. The web-based SSAPredict tool [5] can purportedly predict the fastest SSA to use for a given model via topological network property analysis. As will be seen, it is not always correct. According to Sanassy et al. (2015) [5], despite being one of the top 4 algorithms out of the 9, Tau Leaping still performed worse than other algorithms on many occasions. However, Tau Leaping often far outperforms other algorithms for economy of time (see Section 3) and reportedly has better performance at higher reaction and species graph densities. Thus, SSAPredict should only be treated as a guideline for the best algorithm, especially given its incorrect recommendations as seen in Section 3.

### 1.3. Biological Multicellular Characterization In Silico and Otherwise

With regards to the mechanisms underlying multicellular simulation, it is apparent that small cellular phenotypic changes can have significant biological implications [2]. The outcome of understanding the genetic and phenotypic properties of cells is the ability to mechanically predict their emergent consequences. Cellular and subcellular phenotypic phenomena can be derived from a variety of multicellular and biological literature sources [2,16,17,18,19,20,21,22,23,24] and operate in conjunction with extracellular characteristics [10,16,17,19,25,26] to produce emergent consequences such as cell sorting [9,26], morphogenesis [9], patterning [26], fitness [27] and many more. As a reassurance to modelers, it was observed that the emergent phenomena list was far more extensive than the fundamental cellular and extracellular phenomena from which they emerged, although the permutations, including spatial organizations, are innumerable.

Cellular and subcellular phenomena may include metabolic and gene regulatory networks, cellular morphology [2] and viscoelasticity [17], receptors [18], adhesion molecules [19], cellular polarity [20], membrane evagination/protrusions/projections [16], membrane invaginations, phagocytosis [16], endocytosis [21], exocytosis, secretions [19,22] fluorescence [23], motility [19] and contraction [24].

In addition to the cellular phenotype, extracellular considerations must be factored in, whether or not they arise from the phenotype of local cells. These include diffusion and degradation of chemical concentrations, the extracellular matrix [17] (synthetic [16] or otherwise), extracellular polymeric substances [10], contact signals, obstacles and surfaces, hydrodynamics [10,25], gravity [19], pH dynamics and thermodynamics [10], extracellular reactions [26] (e.g., digestive). These phenomena can have relevance to disease states, just as an example the inflow and outflow of aqueous humor in the development of acute closed angle glaucoma, which influences histological pressure (specifically intraocular pressure) [28].

The combined consequence of cell level and extracellular phenomena are emergent consequences such as cell sorting [9,26], phenotype switching and determination [27], necrosis [11], cellular decay [10] and death [9], differentiation [9], undifferentiation [24], carcinogenesis [29], morphogenesis [9], patterning [26], fitness [27], symbiosis and synergism [30], competition [25], cross-feeding [31], ecosystems, migration [16,23] and chemotaxis [31], population level viscoelastic effects and deformations [17], metastasis [16], population growth and regulation of growth [20], apoptosis [16,22], cellular boundaries [32] and edge detection [23], population heterogeneity [33], attachment [25], detachment and reattachment of agents [10,27], signal sinks [34]. degradation of extracellular materials, including the extracellular matrix [11], immune responses, vascular behavior (e.g., anastomosis [26], angiogenesis [30], extravasation [35]), electrophysiology [4], cell sloughing [29] including of cancer cells, tortuosity [2], intelligence and synapses [36], tissue/exterior interfaces [2], ligand mediated interactions, drug delivery [35], photoreception [34] and more.

### 1.4. Computational Considerations

High-performance computing and extensive parallelization is not uncommon with multicellular simulation [10,11,26]. Other computational enhancements include the clustering of similar cells phenotypically [27], referred to as ‘super-individuals’ [10], perhaps based on the assumed similarity of the local biochemical microenvironment [11,31], or into nearest neighbor lists by proximity [10], use of state outputs with subsequent external visualization [10,11] following “batch processing” [22], as opposed to real-time approaches [19,23], GPU and CPU parallelization [11,22,36] with as few as one cell per CPU, random update ordering [27] to remove bias, Voronoi tessellations to abstract spatial distributions [29], graphical merging of objects [30], client–server architecture [22] and the use of small scale representations of a functionally identical larger system [31]. Domain-based computing is an essential hallmark of multicellular simulation tractability and computation, allowing for parallelization, as well as structural and functional discretization. Also, with the need to consider multiscale phenomena, multiple timesteps are often used, referred to as a “pseudo steady-state approximation”, because temporal resolutions may be different enough that certain processes are “frozen” during those smaller time steps [10,37].

The initialization of spatial configurations, or what might be considered ‘bioprinting’ in silico, can allow for proportionally distributed heterogenous populations, for example in the cortical layers of neurological tissue simulations [36], thereby bypassing stages of developmental emergence. Initial simulated arrangements of cells [19] as well as model generation [38,39,40] have also been attempted using micrographs. Multicellular states, emergent or otherwise, could be saved and experimented on in silico [2] and manipulated by playback controls [22].

### 1.5. Pre-Existing Multicellular Simulation Methodologies

An on-lattice [9] approach refers to a spatially discretized space, where only the discretized spaces of fixed resolution can be occupied. Off-lattice refers [11] to less defined increments of space, for example 3D localization at floating point precision, often using an agent/individual based approach. That said, hybrid methods are common, for example diffusion is often represented through voxel discretization [10,19,41] in otherwise agent-based solutions, providing for fine and even spatial control. Some solutions are entirely on-lattice [9], notably the Cellular Potts method, which was described as an Ising lattice [42], utilizing ‘index-copy’ occurrences via Monte-Carlo Metropolis dynamics method with Boltzmann acceptance [9]. Lattice approaches tend to be more morphologically manipulable due to total discretization, but with inevitable computational costs. Cellular Potts (aka. Glazier-Graner-Hogeweg) multicellular simulators include, perhaps most convincingly, CompuCell3D [9]. Vertex approaches can take on a nodal form in the case of the Finite Element Method, with the discretization of a body into nodes on a mesh to solve complex problems utilizing degrees of freedom. An example using a “subcellular element model” with nodal meshes was the multicellular EmbryoMaker [24] solution, which alluded to an apparently computationally expensive yet high resolution solution with significant morphological flexibility. A hybrid Finite-Element Cellular Potts approach is in VirtualLeaf [43]. Agent-based multicellular simulators, almost always hybridized with a domain-based discretized layer or possibly other modalities, are apparently the most abundant, e.g., Gro [23], BSim [18], Simbiotics [19], NUFEB [10], CellModeller [34], iDynomics [27], Biocellion [26], PhysiCell [11], EPISIM [2], SimuLife [30], Cell Studio [22] and MecaGen [42]. Some, such as Chaste [4], offered hybrid solutions, such as both on and off lattice modalities. Smoldyn 2.1 [44] spatial stochastic simulator possessed reaction-diffusion-compartmentalization capabilities.

## 2. Materials and Methods

**Figure 1:**
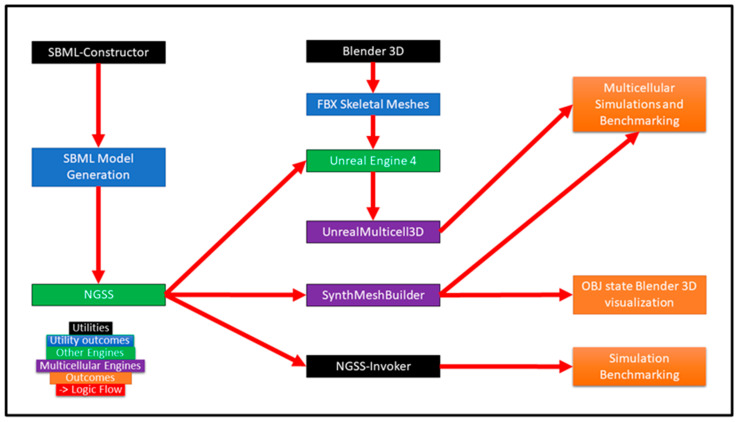
A novel workflow was developed involving utilities, backend components/engines, as well as two multicellular spatiotemporal methodologies featuring different technical approaches. Modularity could be considered across the workflow and/or at the algorithmic level.

The overall methodology can be seen in Figure 1. The benchmarking of two novel 3D multicellular simulators with and without NGSS integration on a high-end personal computing system will be described to demonstrate computational limits, reveal enhancements and demonstrate the scalabilities of different approaches. A specific NGSS version was tailored for Windows and its integration with multicellular layers was pursued to bridge the gap between multicellular simulation and Synthetic Biology CAD design. This methodology would provide insights into CAD considerations regarding simulation architecture, ergonomics, and demonstrated principled in silico population level emergence from the algorithms.

SBML-Constructor was a utility developed to automatically generate simple SBML format biological reaction pathways of differing homogeneity, lengths and topologies for benchmarking with the NGSS stochastic simulator coupled to a multicellular simulation layer. It was developed using SBML documentation as formatting guidance [45] to overcome interoperability issues [6].

NGSS-Invoker was a simple utility program developed to execute and benchmark an adapted Windows version of NGSS multiple times and hence fully saturate the CPU to measure the time taken for a user defined number of NGSS activations to complete.

### 2.1. UnrealMulticell3D

UnrealMulticell3D (UM3D) is a prototype, agent-based, off-lattice, real-time, 3D multicellular simulation software developed in Epic Games’ Unreal Engine 4 (UE4) and C++. A version is available via GitHub [46]. UM3D addressed inferior graphical solutions [19,23] with the state of the art 3D UE4 used for blockbuster gaming productions. UE4 performs physics calculations utilizing PhysX which has the potential to utilize GeForce GPUs to perform Newtonian physics, although UE4 apparently only harness its CPU implementations (see Section 3.3.2). UM3D also addressed the lack of real time ergonomic user interfaces and Windows accessibility compared to otherwise very robust methods [10,11].

**Figure 2:**
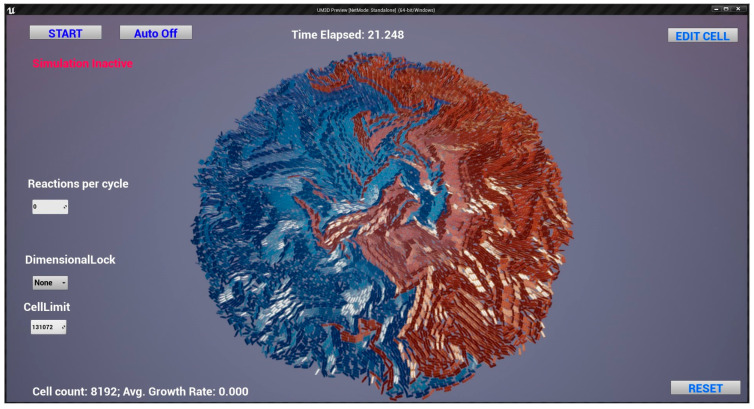
UnrealMulticell3D (UM3D) dimensionally constrained monolayer colony of bacillus cells. See also supplementary Video S1 for more UM3D capabilities and dynamic visuals.

Figure 2 demonstrates a dimensionally constrained bacillus colony monolayer that, in this case, took approximately 21.248 s to reach 8192 cells from a single cell, as measured by the epoch-based timer. Circular, raised bacillus colony morphology was used for benchmarking purposes, which was the physical outcome of proliferation and growth interacting with the Newtonian physics native to UE4, thus reflecting the state of software parameterization at that time. Note the emphasis on emergent colony behavior as a result of individual cell parameterization. See supplementary Video S1 for other example morphologies and parameter settings. As will be discussed subsequently, additional biophysically influential phenotypic traits were layered upon this initial design.

Cellular assets were produced through 3D skeletal mesh designs in Blender 2.90.1, including the use of shape key animations for cellular cleavage during division. The file format for transfer to UE4 was FBX [47]. FBX is a popular file format for conveniently interchanging rigs, meshes, animations and texture information between 3D graphics software. The basis mesh consisted of 3551 vertices for a bacillus shaped cell, but a simplified mesh was successfully trialed using only 8 vertices with only visual implications since physical interactions used a ‘Capsule Component’, inherent to the UE4 ‘Character’ blueprint class. The bacteria in UM3D would split upon reaching a shape key setting of double the size, from 1.5 microns to 3 microns in length in the case of bacilli, based in the simulation literature [19]. UE4 use has the capacity to overcome limitations in the modelling of morphology and heterogeneity compared to the most promising agent-based multicellular solutions [10,11]. Also, such solutions lack interdisciplinary ease of use, with the literature recommending the harnessing of graphical user interfaces [14] as exhibited in UM3D. In fact, a modern alternative to standard programming, in the form of visual scripting, has been harnessed within industry such as the “design of experiments” company Synthace [48], who reportedly coordinated with AstraZeneca, but also occurs to an extent in various Synthetic Biology tools [1,6,8] through drag and drop designs. Both Blender and UE4 offer such capabilities, and these were exploited particularly in UE4. Such scripting methodologies allow for fast, reliable, ergonomic designs; undoubtedly contributing to the great successes of tools that exploit these methods.

**Figure 3:**
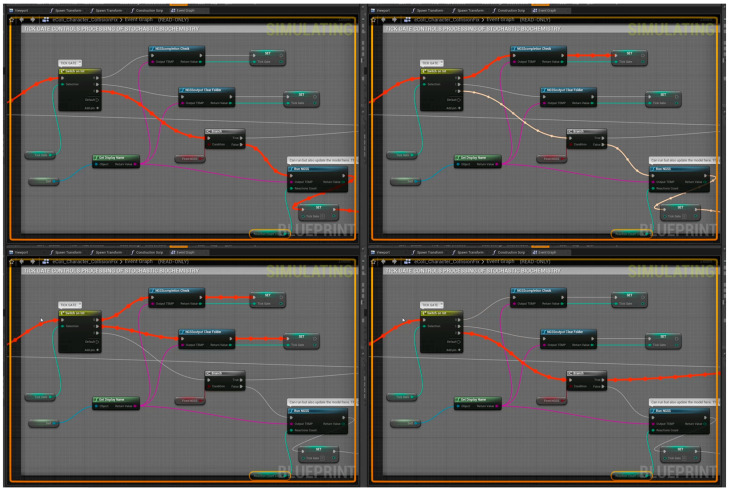
Control Flow featuring NGSS integration into UM3D using a custom C++ blueprint function library. Blueprinting was also used for other cellular computations/functions. **Top Left**: The construction script defaults Tick Gate to 2, allowing the biochemical model to be fired. Tick Gate is set to 0. **Top Right**: NGSS completion is awaited and cellular functions besides physics are arrested. Tick Gate is set to 1 when the output file is detected. **Lower Left**: NGSS output file is cleared, and deletion is confirmed. Tick Gate is set to 2. **Lower Right**: The Boolean value of “FiredNGSS” is true so the “Run NGSS” node is bypassed allowing for growth, division and other phenotypic computations on the agent cell. In this prototype the biochemical model was fired only once per cell cycle, hence daughter cells would undergo the same process.

Within Unreal Engine 4, “Blueprints” were harnessed, which is the node based programmatic method available to UE4 developers. The conditional cellular logistics operate on the “Tick” event that was set to fire every frame on an agent by agent basis. Each agent would run a copy of this blueprint. The “Tick Gate”, as it was referred to during development, is a blueprint control structure utilizing the C++ blueprint function library that was written specifically for the integration of the NGSS stochastic simulator into UnrealMulticell3D. The “Tick Gate” is elucidated in Figure 3 (see also supplementary Video S2).

Additionally, an ergonomic cell editor was designed that would permit the manipulation of rates and parameters influencing proliferation, contact inhibition, cell death, neoplastic mutation, cell to cell adhesion, differentiation, cell shape (coccus or bacillus), motility force and the inheritance of color (fluorescence/pigmentation). To contrast with potential biological features (see Section 1.3). It should be evident that proliferation rate, adhesion, differentiation, morphology, motility, and even color, are fundamental to the nature of a cell’s behavior, and hence immediately necessary to model. However, phenotypic parameter modulation would likely be best described through bioregulatory, metabolic and signaling models. The means by which regulatory models could be simulated were discussed in Section 1.1. For example, division at twice the cell volume would be insufficient to describe mitosis of the zygote in the formation of a blastomere, which involves no volumetric change [49]. During these extremely rapid divisions the G_1_ and G_2_ cell cycle phases are reportedly absent; with elevated concentrations of MPF (maturation promoting factor) being a key regulator of cyclical mitotic events as per the Tyson’s Cell Model [50] available on BioModels [51]. Indeed, this model was utilized by the EpiSim multicellular simulator [13]. Assuming accuracy and utility of Tyson’s Cell Model, the multicellular layer (e.g., UM3D) would need to be adapted to accommodate these division processes, respecting collision, physical demarcations and presumably individual logical processing for each daughter cell, since computation as a synchronized, homogeneous population would be inappropriate for developmental biology and subsequent differentiation unless population groupings could be solved together as clusters. A major challenge for accurate multicellular biophysical simulations is cellular morphology, achieved here only for two types of cell/collision shapes. The challenge is not only the mesh visualizations, but also the viscoelastic variations, cytoskeletal modulation of shape and accurate modelling of collision. Not to mention the computational expense complex shapes might require without abstraction, and the potential requirement to represent cell polarity.

Nevertheless, within UM3D initial conditions could be set pending emergent outcomes. These parameters could be set to vary randomly through the simulation run, giving rise to daughter cells with random properties/characteristics. Clearly, tight parametric control and regulation would be needed to produce desired geometrical and functional cellular configurations, perhaps machine learned and/or evolutionarily selected [52]. With appropriate parameterization it can be envisaged that evolutionary mechanisms (e.g., heuristic genetic algorithmics) could be applied to such a system towards single-objective [3] or multi-objective [52,53,54] optimization. Stochasticity could be introduced at various levels, not least using NGSS, however in this prototype the primary noise was generated by slight variations in cell orientation following division. 

Showcasing high-throughput morphogenesis, in UM3D, a cell limit could be set and parameters could be randomized with an infinite loop of automatic simulation cycles, albeit with temporally determined measures (e.g., simulation reset) taken in the case of limited proliferation (i.e., simulation stagnation). Other parameters for experimental reset might be considered. The main focus was proliferation from a single cell; however, a lattice of cells could also be initialized and even spun stochastically or placed within a prototype scaffold. As already noted, such strategies as heterogenous cellular distributions were used for appropriate histological functionalization in silico [36]. Eventually, to improve on the system in Figure 3 and reduce the latency of file I/O, a client–server architecture was prototyped between a C++ client and UM3D to run an oscillatory sine function for single cell bioluminescence (beware flashing images, supplementary Video S3). For this a TCP socket connection was made using the UE4 “TCP Socket Plugin” by SpartanTools. The above characteristics of UM3D could be considered for improvement in future installments. Subsequent progress would also require the modulation of phenotype with biophysical ramifications via specific stochastic simulation models.

### 2.2. SynthMeshBuilder

**Figure 4:**
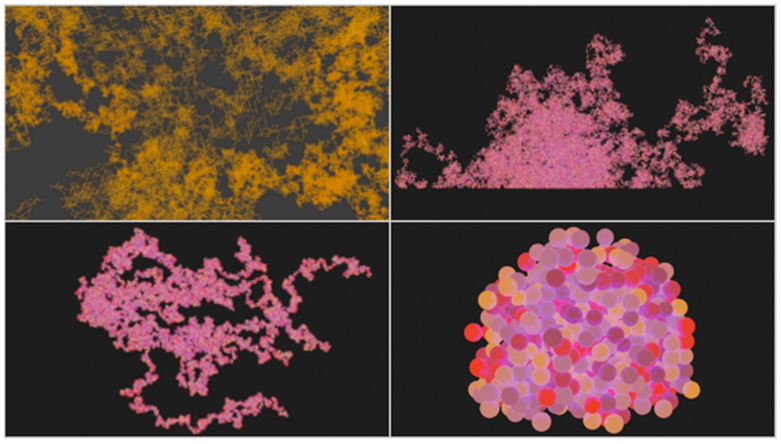
SynthMeshBuilder’s diverse morphology generation of mesh-based cell networks (**upper left**), highly scalable one million cell colonies (**upper right**), parallelized “stochastic chain extension” reminiscent of staphylococcus clusters [55] used for benchmarking (**lower left**) and an alternative on-lattice algorithm with random update order (**lower right**), as visualized with Blender (see supplementary Video S4).

SynthMeshBuilder (SMB) is a procedural, multithreaded, vertex-based, batch processed, 3D multicellular mesh generation prototype software, developed in .NET Core 3.1 C# for Windows, blurring the line between On-Lattice and Off-Lattice approaches, with an agent-based character and utilizing generative rules-based decision making. Mesh generation is practical since vertices can be represented by minimal data and meshes can be used to construct and regulate large objects such as tissues. SMB harbors similarity to a ‘family’ of multicellular tools [10,11,26,27]. Commonalities include batch processing, retrospective visualization, spherical agents, proliferation focused, computationally parallelized, highly scalable, independent solutions; although SMB has not yet utilized HPC. Batch processing can be recommended as it negates the need for live rendering costs, and can provide for interface-free computation if embedded in a suite. Animation of growth was possible in SMB due to the ordered sequence of vertices in the OBJ output file that Blender 2.90.1 could iterate over with particles (Figure 4).

SMB was trialed with various approaches, including on and off lattice solutions. The “Pseudo Off-Lattice” algorithm used no fixed lattice boundaries but placed vertices at unit distance from the parent and was used for benchmarking. Within this Pseudo Off-Lattice approach, the “Stochastic Chain Extension” ruleset (see supplementary Videos S4 and S5) insighted the most recently generated cell on a computational thread to replicate in a random direction into an unoccupied location. Because the distribution was effectively on lattice due to unit spacing, the exact coordinate could be checked for the presence of a vertex without explicitly checking for a geometrical overlap based on cellular diameters. “Tunnelling” of cells was used if the cell could find no free space to expand into locally. 

CPU multithreading was achieved using System. Threading, with an array of threads defined by Threads[x] = new Thread(() => BuildMesh(index, CellsPerThread, “Parallel”)), where index denoted the thread index and given Threads[x].Priority = ThreadPriority.Highest. Inside BuildMesh(), each thread could create NGSS processes with different input commands. Next, random displacement would be performed. The random displacement, before tunnelling, in each plane was calculated as reflected by the following pseudocode: *X, Y or Z displacement = Random(0 to 2) − 1*. If the coordinate reached via the vector (X,Y,Z) was occupied, tunnelling would be calculated by multiplying each element of the displacement vector by an increment with procedural checks within a while loop until a free space was found displacements (X,Y,Z) = (X*increment, Y*increment, Z*increment), where X, Y and Z represent displacements between −1 and 1 generated as aforementioned. Putting constraints on axes could simulate uniform surface obstacles (Figure 4 lower left and supplementary Video S5). 

An alternative On-Lattice approach was decisively On-Lattice since it utilized a fixed sized 3 dimensional array representation of the world space. An initial cell would be seeded at the (0,0,0) coordinate. The matrix would then be scanned iteratively, and given the existence of a cell there would be a probability of the initial cell seeding another. A Monte Carlo (random update ordering) approach was eventually implemented rather than iteratively scanning the array sequentially. This could be performed by operating on all elements of an integer array in a given spatial dimension using the following C# code: Enumerable.Range(0, WorldDiameter).OrderBy(c => random.Next()).ToArray() to generate a pseudorandom reordering to iterate through.

OBJ encoding of edges occurred between parent and daughter cells/vertices to generate the histological mesh. Since each thread was given a target population to reach, vertex and edge OBJ encodings would be positioned at indices on a string array calculated by (x + CellsPerThread_temp * ThreadID_temp), where x was the replicative iteration by the thread, thereby avoiding any memory access errors. In multicellular scenarios such bonds represented by these edges may be formed by extracellular substances, cell-matrix adhesion and cell-cell adhesion. Such interwoven webs could be used for intercellular communication simulations. High entropy stochastic outcomes can be progressed towards low entropy organization and behavior by increasing algorithmic control. The benchmarked colony formations were morphologically different from those of UM3D due to the underlying algorithmic differences.

**Figure 5:**
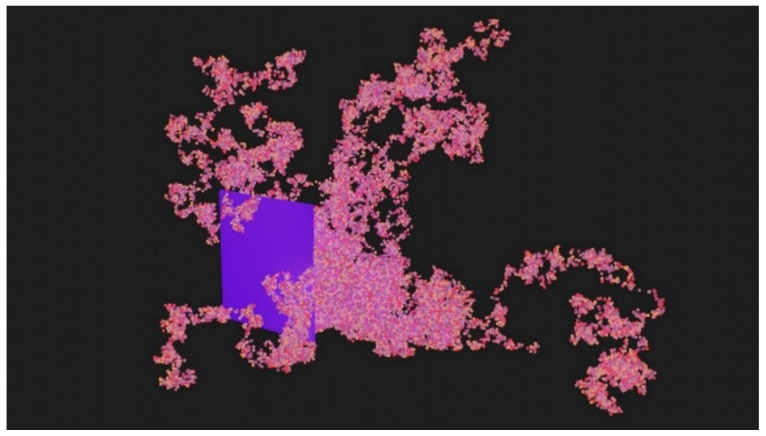
600,000 “Pseudo Off-Lattice” cells clustering around an obstacle as visualized in Blender via .OBJ files output by SynthMeshBuilder. The stochastic expansion of the colony is evident from the morphology. Collision surfaces of such a nature could also be used to trigger contact signaling calculations.

An obstacle simulation was also performed with 600,000 cells using 10 threads and 60,000 cells per thread. Figure 5 depicts these cells swarming around a thin barrier as visualized using Blender. The proliferating cells would interact with vertices from a secondary mesh, thereby preventing their positioning inside the obstacle. Adhesion and juxtacrine/mechanosensory [56] regulatory signals could be imposed on simulated cellular behavior by collision surfaces in silico with relevance to tissue engineering scaffolds and extracellular matrices.

### 2.3. NGSS Use via NGSS-Invoker, UnrealMulticell3D and SynthMeshBuilder

No CRN/regulatory model feedback into the simulators was attempted or achieved when running the external NGSS metabolic simulator in relation to the multicellular simulation layers (SMB/UM3D) besides NGSS completion checks via CSV output file IO between executions. To progress this in the future requires strong biological justification, and multicellular simulation studies had only modestly experimented with this, such as the use of Tyson’s cell cycle model [13]. A recent study utilized the iAF1260b model [57], and came from a Whole Cell Modelling perspective, combining stochastic gene expression and flux balance. Whether it is through stochastic simulation or another solution such as the use of Boolean networks [29], flux balance analysis [31], deterministic or hybrid algorithms [14] subcellular “decision making” affecting cellular phenotypes is vital in order to elicit regulated, emergent multicellular behavior in silico. However, for prototyping and benchmarking purposes it was sufficient to execute the control flow without phenotypic or genotypic changes via feedback.

The control flow for awaiting NGSS completion in SMB was similar to UnrealMulticell3D in terms of awaiting output .csv confirmation. Inducing threads to sleep between completion checks reduced the CPU demand within the thread file-checking while loops thereupon increasing tractability. The duration of thread sleeping could undergo optimization efforts, however undoubtedly more ideal inter-process communication can be sought, such as via network sockets as prototyped with UM3D.

## 3. Results

### 3.1. Hardware, Software and Models Used for Benchmarking

Benchmarking was performed using a G7 7700 Dell Laptop, Intel(R) Core(TM) i7-10750H CPU @ 2.60 GHz (6 cores, 12 logical cores) processor, 16.0 GB RAM, NVIDIA GeForce RTX 2060 6 GB VRAM graphics card on Windows 10 64-bit.

SBML-Constructor was used to produce sets of arbitrary, benchmarkable SBML CRN kinetic models with serial pathways up to 128 reactions in length with low enzyme and high substrate concentrations to homogenize performance. Two sets of models were generated, a set with separate enzymes (multi-enz) for each reaction and one with a single enzyme (single-enz) mediating every reaction. Most SBML models from the BioModels database [51] were reported as 50 reactions or less [5], with a few having as many as 1800 reactions. Thus, the range generated by SBML-Constructor, up to 128 reactions, could give a reasonable sense of tractability for models from curated model archives. NGSS-Invoker was used to benchmark NGSS without spatial simulation using the SBML models. UM3D and SMB engines were benchmarked with and without NGSS, with spatial and graphical consequences but without actual logical feedback from the SBML model itself.

### 3.2. Benchmarking without the Multicellular Layer

#### 3.2.1. Single Cell Performance via NGSS-Invoker

Initially, three different Stochastic Simulation Algorithms (SSAs) were tested based on qualitative experience of their temporal performances; Tau Leaping, Direct Method and the Next Reaction Method. A fourth was tested retrospectively on recommendation by the SSAPredict tool [5]: the Logarithmic Direct Method. With the multienzyme model, SSAPredict concluded for the 4, 8, 32, 64 and 128 reaction models that the Logarithmic Direct Method should be optimal. For 2 and 3 pathway reactions the Optimized Direct Method was recommended. Nevertheless, Tau Leaping performed faster, likely because it favors low propensity (slow, low probability) scenarios [15] matching the models generated. For all algorithms, time to complete increased with a polynomial trend as the number of reaction steps to complete increased. Up to 6 reactions the Tau Leaping algorithm possessed a nuance where it followed the relatively slow Direct Method.

**Figure 6:**
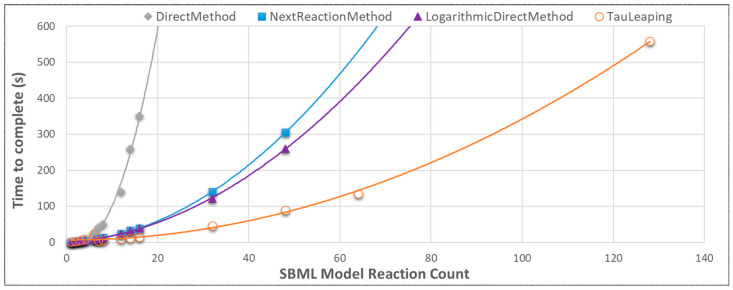
For the low propensity models generated by SBML-Constructor, the Tau Leaping NGSS algorithm performed best using the multi-enz models. Results were generated using NGSS-Invoker. Optimized Direct Method is not shown as it was suggested retrospectively to initial benchmarking and only recommended for pathways of 2 and 3 reactions. Likely it was immediately discarded due to inferior performance and practical time constraints. SSAPredict was shown to be wrong with its prediction of performance for the Logarithmic Direct Method, i.e., Tau Leaping was 2.29 times faster than the Logarithmic Direct Method with the 48 reactions model.

A comparison of the behavior of different SSA algorithms was also performed for the Single Enzyme model set, where NGSS performed identically in every case despite SSAPredict recommending the Logarithmic Direct Method (LDM) and the Partial Propensity Direct (PPD) Method. The single-enz topology was far slower than multi-enz. Out of the conditions tested, NGSS turnover was deemed fastest under Tau Leaping, interval 0.1, MAX_TIME 3, with multiple enzyme models over a single run (Figure 6).

#### 3.2.2. Multicellular Performance via NGSS-Invoker

**Figure 7:**
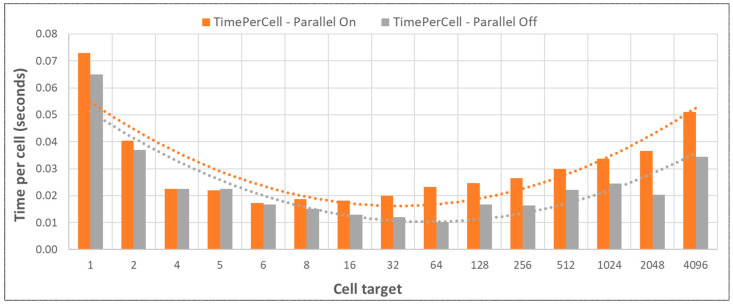
Towards processor saturation caused by increasing “concurrent” cell target computations of NGSS activations, time “per cell” for completion of the reaction model decreased. Beyond saturation and towards cell completions, hence more .CSV file checking, time per cell increased gradually (note the uneven unit distributions on the *x*-axis). The above data used the NGSS settings determined in the previous section. Minimizing IO in the future would be ideal. These results were generated using the in-house NGSS-Invoker utility coupled with NGSS executions.

The fastest performing settings were brought forward and NGSS-Invoker was used to saturate the CPU with multiple concurrent NGSS process activations. Because only a single run was being made, the ‘parallel on’ NGSS setting served no purpose, but behaved differently from the ‘parallel off’ setting. Note that the NGSS parallel thread setting is independent from the parallelization by multiple NGSS activations and is used to average multiple stochastic runs. As the “cell target” (NGSS completions) increased, initial performance increase towards plateau was due to the concurrency of NGSS activations on the processor, reducing time per cell (Figure 7). This was due to processor saturation, hence full CPU usage across all logical cores. The conclusion that was brought forward was that for a single run, the ‘parallel off’ NGSS setting was the less time consuming algorithm.

### 3.3. Benchmarking with the Multicellular Layer

#### 3.3.1. Multicellular Performance via UnrealMulticell3D with NGSS

**Figure 8:**
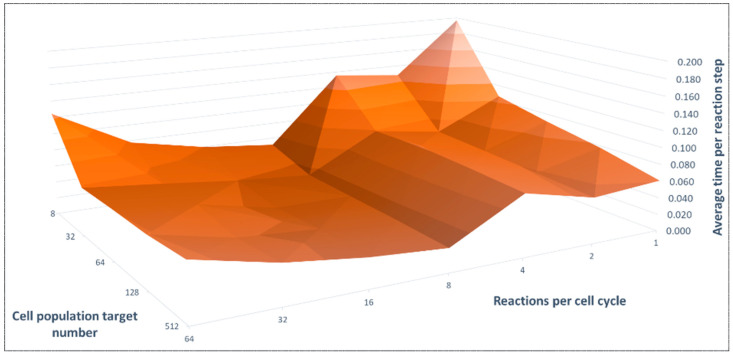
Benchmarked time performance data subset of UnrealMulticell3D on a time per reaction step basis. Note that greater cell target populations ensured processor saturation, explaining the peaks, with performance consistency upon saturation given unchanging reactions per cell cycle (RPC). There was polynomial scaling with RPC beyond the NGSS nuance up to ~7 RPC.

Using the conditions established from the previous experiments, NGSS could be run in the spatial multicellular simulators once per mitotic cell cycle and the reaction count could be varied by changing the multi-enz SBML model. Here we discuss this implementation into UM3D. The three variables (time, population, reactions) resulted in 3D statistical data (Figure 8). Starting from a single cell, the duration to reach a given population size was longer for larger cell target populations and as the number of reactions per cell cycle was increased. Polynomial time scaling with reactions per cycle induced by the NGSS algorithm was likely because the reactions in the model were not mutually exclusive and, hence, had computational interference, matching NGSS behavior. The optimum number of reactions in the model was 8 due to the nuances of the Tau Leaping algorithm and the range of models tested (note that 7 reactions was not tested). On the other hand, scaling towards a target population was essentially linear given constant reactions per cycle. A linear scaling was unsurprising as increasing the cell population target simply increased the number of repetitions of the same action, especially once processor saturation was reached.

#### 3.3.2. Multicellular Performance via UnrealMulticell3D without NGSS

**Figure 9:**
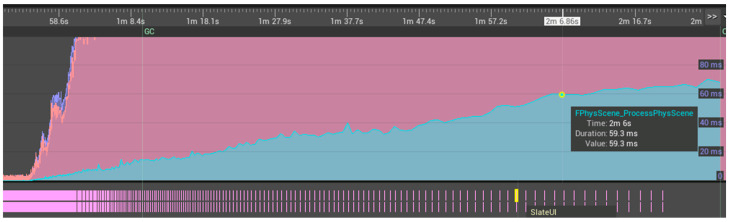
A performance profile was run for UM3D using the Unreal Insights utility with the camera turned off. FPhysScene_ProcessPhysScene (blue), processed on the CPU, required greater processing as the cell population expanded. The plots behind the blue plot can be ignored for this discussion. The lower track illustrated limited GPU activity involving intermittent SlateUI updates. PhysX calculations appear to be restricted to the CPU by UE4. The impact of physics processing on the CPU might have been missed at the relatively low cell populations tested (up to 4096 over a span of ~20 s) because of its fairly modest initial CPU usage.

**Figure 10:**
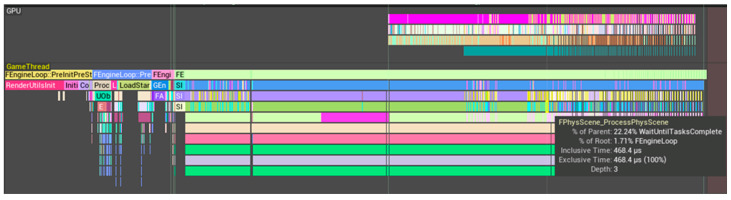
Unlike when the camera was off (see Figure 9), the GPU had a vast assortment of functions to fulfill when the camera was turned on (GPU track, top). However, none of the functions appeared to perform physics calculations; whereas on the CPU GameThread track, FPhysScene_ProcessPhysScene continued to be processed.

Starting from a single cell, target populations were reached over a measured time without NGSS processing to evaluate the behavior of the multicellular simulation layer alone. Physics was compared in the on and off states with increasing model sizes. Both cases performed undiscernibly, implying that physics computations played little part in overall performance. This led to the hypothesis that Unreal Engine 4 was using PhysX to calculate physics on the GPU. Thus, toggling physics was assumed to have no statistical impact because the CPU was equally saturated whilst the GPU apparently remained unsaturated. On further investigation it appears that this assumption was false. Profiling GPU and CPU behavior with the Unreal Insights tool (with the ‘in game’ command Trace.Start default, gpu) revealed that there was minimal activity by the GPU when the renderer was off during simulations, apparently only performing basic user interface processing via SlateUI. The Slate UI Framework is Unreal Engine’s cross-platform user interface framework [58,59]. In fact, the only evidence of physics processing was on the CPU (Figure 9 and Figure 10) via FPhysScene_ProcessPhysScene, with FPhysScene documented as involved with PhysX processing [60].

The GPU operated on a much greater range of functions with the camera on (Figure 10), however the CPU GameThread demonstrated the only evidence of physics operations. In combination these are strong indicators that physics was not performed on the GPU and that this version (at least) of Unreal Engine 4 reserves the GPU primarily for graphics rendering rather than GPGPU (general-purpose computing on graphics processing units) implementations, an apparent limitation of standard hardware architectures and UE4. Nevertheless, the prospect offered by physics libraries still stands, with Classical Mechanics resolvable through GPGPU implementations for multicellular simulations of the future.

A benchmarking effort was also made to assess other factors such as the impact of cell textures, with no discernible computational costs. That said, the performance enhancement from turning the camera away from the cells was dramatic, demonstrating a significant slowdown associated with rendering costs, not only on the GPU but potentially including activity on the CPU “Rendering Thread”. This was apparently not due to graphical and GPGPU physics operations competing for resources on the GPU (Figure 9 and Figure 10). A batch processing mode accompanied by the export of simulation states could be conceived of. In fact, this information led to the development of a camera on/off toggle on keypress in subsequent developments that was retrospectively used to achieve the benchmarks in Figure 9 and Figure 10. The removal of an aesthetic animation shape key for the bacterial cleavage site had no impact on the simulation time, but simplifying the bacterial mesh from 3551 vertices down to eight vertices significantly sped up the simulation, particularly at higher cell populations. The physical mechanics were mediated by an unchanged collision capsule. Hence the change in mesh vertex-count would have no impact on the accuracy of the simulation, although it would result in mild visualization artifacts.

**Figure 11:**
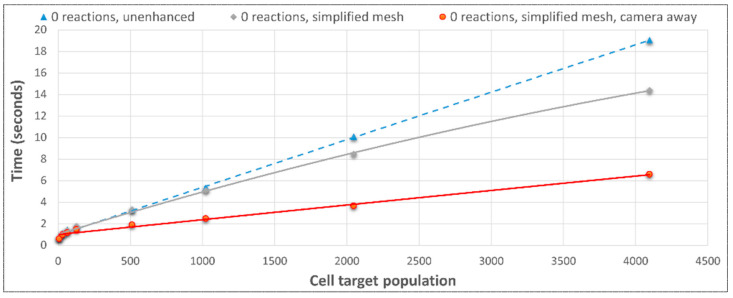
Performance enhancement of UnrealMulticell3D without NGSS, due primarily to a move towards batch-processing and reduced rendering costs. Mesh simplification enhanced performance to generate 4096 cells by 1.33 times. Combined mesh simplification and camera redirection enhanced generation of 4096 cells by 2.63 times.

**Figure 12:**
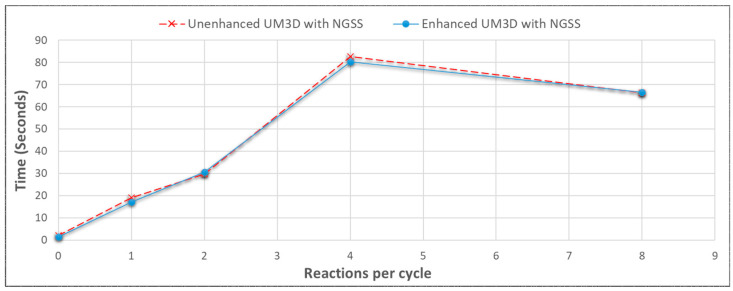
Despite enhancements to the UnrealMulticell3D base engine depicted in Figure 10, performance with NGSS was identical, demonstrating that NGSS was the limiting factor with respect to performance. The above depicts an average time across all benchmarked cell target populations for each reaction per cycle pathway model, used here as an indicator of the average performance.

The impact of the collective performance enhancements was compared to the conditions prior to benchmarking (Figure 11). Regardless of these significant baseline engine enhancements, use with NGSS had very limited to no benefit since NGSS was the limiting factor in the total simulation time (Figure 12), justifying the future pursuit of HPC and/or client–server architecture.

#### 3.3.3. Multicellular Performance via SynthMeshBuilder without NGSS

**Figure 13:**
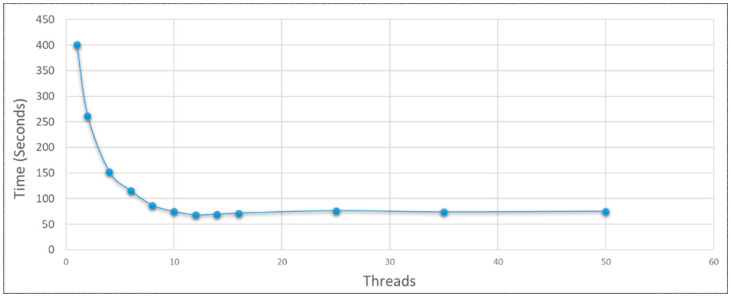
Mesh generation by SynthMeshBuilder to a 252,000 vertex population was temporally enhanced up to CPU logical core saturation (12 threads) when parallelized with various numbers of launched threads demonstrating successful multithreading on the CPU architecture.

The first benchmarking experiment with SMB targeted 252,000 cells. The number of threads ranged from 1 to 50, with cells per thread ranging from 252,000 to 5040 respectively. Performance plateaued as threads reached the number of logical cores (12) beyond which there was a very slight decrease in performance (Figure 13). 10 threads were brought forward for benchmarking to maintain a responsive UI and operating system. Linear scaling was not achieved as the number of cells increased, rather there was a polynomial increase in simulation time. This is almost certainly because as the cell population grew, the overlap checks on proliferation also grew in number since all cell coordinates were iterated over. Thus, many unnecessary points were scanned as the point-cloud developed. This is where domain-based parallelization or nearest neighbor lists [10,19] could be considered, with a reduction of cell by cell processing and thereby the linearization of the trend. An On-Lattice approach with local scanning across the restricted lattice geometry is one option, but Off-Lattice provides for more diverse spatial potential going forward. Alternatively, distinct cell populations could be computed on separate processors within a heterogenous pool of cells. The probabilistic “bridge” concept between physically isolated populations might also be considered [22].

#### 3.3.4. SynthMeshBuilder vs. UnrealMulticell3D Performances with NGSS

**Figure 14:**
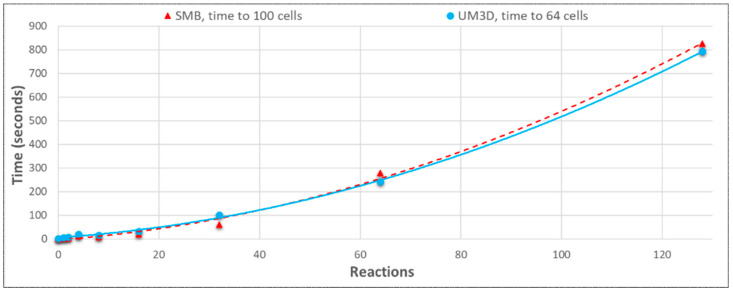
Both coupled with NGSS, SynthMeshBuilder scaled faster than UnrealMulticell3D due to its streamlined algorithmics, but with a similar trend. The target populations differ above.

**Figure 15:**
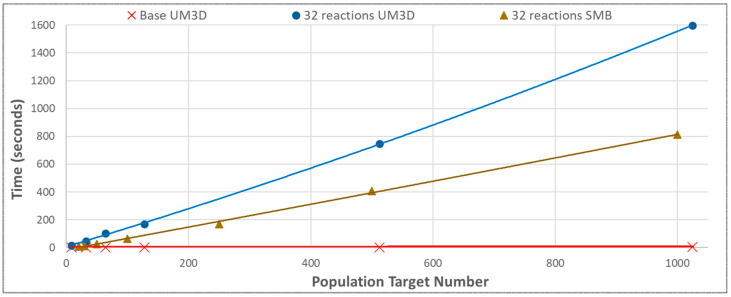
The base multicellular simulation layers could generate 1000-cell populations within seconds (UM3D) or fractions of a second (SMB). However, adding a moderately sized reaction network (32 reaction steps) with parallel NGSS processes (one for each cell cycle) resulted in a drastically more time consuming performance profile operating on the order of several minutes to complete. Because NGSS could use as much as a single core (two threads) of processing power for each activation, by the time only 6 cells that reached the processor could be saturated, giving an overall linear scaling as NGSS processes were queued for completion on the rapidly saturated single processor. The same linear scaling would be expected with HPC but with a shallower gradient, at least once the HPC cores were fully saturated. For example, with the 32 reaction network UM3D proliferated 299 times slower to 1024 cells than without it (1598.20 s vs. 5.34 s).

SMB scaled in a similarly polynomial fashion but performed faster than UM3D when NGSS was processing models (Figure 14). This should be attributable to the fact that SMB is algorithmically far less complex than UM3D and was able to leave the majority of the CPU for NGSS to utilize and was entirely batch-processed, thereby circumventing live rendering costs. The scaling followed NGSS behavior for the network sizes. With NGSS, SMB was able to perform almost twice as quickly as UM3D (evidenced in both Figure 14 and Figure 15) due to its simpler ground-up algorithmics, specifically streamlined for NGSS performance. The performances of the multicellular engines with and without NGSS were very different with even modest SBML model conditions imposed, with NGSS lengthening the simulation time dramatically (Figure 15).

#### 3.3.5. SynthMeshBuilder vs. UnrealMulticell3D Scalabilities without NGSS

**Figure 16:**
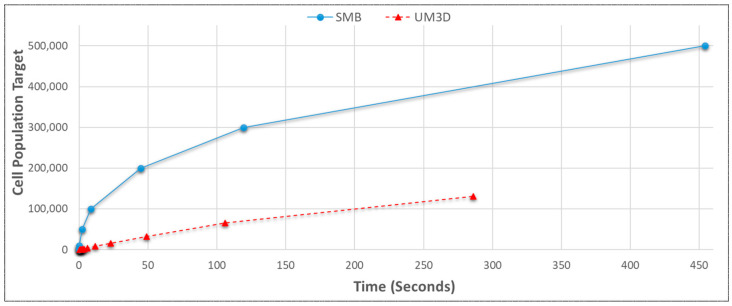
SynthMeshBuilder proved much more scalable without NGSS than UnrealMulticell3D on the personal computing system. By contrast, HPC solutions from the literature could process millions [11] or even tens of millions [10,36] of cells, with thousands [23] or hundreds of thousands [11] reported on modest hardware.

The scalabilities of SMB to UM3D without NGSS were compared (Figure 16). SMB demonstrated far greater scalability (with only 10 of 12 threads) compared to UM3D (with no imposed resource restrictions). UM3D was slower and more unstable, eventually reaching a respectable 131,072 cells. SMB was stopped at 500,000 cells, although it can scale much further. That said, a high cell count is not required for multicellular life. The adult nematode worm Caenorhabditis Elegans, an oft used model organism, has been reported to have as few as 959 cells [61]. However, should regulatory computations (subcellular models) and more multicellular simulation phenomena be added (e.g., diffusion, extracellular agents), scalabilities would become much lower. On the other side of the spectrum, the human retina photoreceptor topography has been reported as being composed of as many as 5.29 million cone and 107.3 million rod cells [62]. Far beyond tractability on conventional hardware without simplification.

## 4. Discussion

Multiple, biologically informed, ground-up approaches to agent-based multicellular simulation were demonstrated; a scalable, prototype, mesh-based, batch processed approach (SMB) and a state-of-the-art 3D engine approach (UM3D). SMB demonstrated that low level abstractions can have scalable yet compelling outcomes reminiscent of classical Cellular Automata, with a reduction of entropic behavior achieved by increasing algorithmic regulatory control. Both SMB and UM3D can benefit from many additional multicellular features (see Section 1.3) that are described in the literature, with some obtainable through open-source code. A critical progression for SMB and UM3D is subcellular model feedback with phenotypic effects, with the choice of subcellular models also of critical importance. Subcellular regulatory models would either be designed or downloaded from a repository. The use of ergonomic graphical user interfaces with powerful features continues to be desirable [9].

The temporal use of physics would need to be carefully considered in order to make multiscale performance accurate, along with the overall careful orchestration of temporality and parameterization in general. The evidence suggests that Unreal Engine 4 performs physics on the CPU with PhysX, despite PhysX being GPU capable, which may reflect a limitation and computational overhead of this live rendered, real-time approach. Also, the results (Figure 11) demonstrated that live rendering had a dramatically negative impact on performance at large scale. Thus, the results alluded strongly towards batch processing methodologies with rendering minimized if rapid performance is desired, such as provided by SMB that harbors similarity to various extant tools [10,11,26,27]. UM3D could be adapted towards batch processing away from real time rendering but while retaining the visualization options. This idea was explored through camera redirection and eventually the addition of a camera ON/OFF toggle. It remains to be seen how the innate limitations of UE4 could be rectified and an exploration of UE5, released 2022, might be worthwhile. Client–server architecture [22] is another possibility that was prototyped in UM3D to overcome some of the performance limitations of local computations and inter-process communication.

NGSS saturation of the processor via process executions demonstrated the limits of a high end desktop computer. Temporal multiscale implications were observed as NGSS’s significant temporal usage contrasted with cell growth, physics and population growth dynamics in the multicellular layer, particularly as seen in UM3D. NGSS has a peculiar performance nuance at fewer than 7 reaction models with the Tau Leaping algorithm, however many reaction networks will likely be larger than 6 reactions. For NGSS, metabolic network topology has a significant impact on performance, as demonstrated by the Single Enzyme models versus the Multienzyme models. It should be noted that stochastic simulator performance might be considerably lower with high propensity reaction models.

Subsequent work should challenge the limits of multiscale, multicellular simulations including the implementation of novel case studies of morphogenetic and functional multicellularity/histology, including subcellular model feedback, better defined heterogenous populations and the continued hybridization of both agent-based and lattice-based (domain-based) modelling. The importance of having identified the key features of multicellular simulation should not be underestimated for subsequent work (Section 1.3), specifically when considering the integration of what has been described as “sub-models” [11]. The connection with this work to the IBW platform for Synthetic Biology in silico prototyping was primarily through the mutual NGSS simulator [6]. Future work could explore the potential of automating the IBL syntax IBW possesses for kinetics models and its hierarchical biological descriptions, which is presently a manual process, similar to the way in which SBML-Constructor exploited the SBML standard format [45].

The current work highlighted just how intensive cell by cell computations are, especially with the use of subcellular biochemical stochastic simulations, and implicated the future use of HPC that might be applied on a cell by cell basis with the splitting of NGSS activations between processors [36] (see Figure 15), or more likely via GPGPU implementations. The ‘first-come-first-serve’ consequences of NGSS activation across processors potentially skews realism, rectifiable primarily by HPC applications of Stochastic Simulation Algorithms (SSAs) and/or a possible Monte Carlo approach. The use of clustering into “super-individuals” [10] can be considered, along with the possibility of population-based or hybrid individual/population approaches. The use of GPGPU algorithms, such as PhysX, is strongly under investigation for subsequent work.

Model verification for multicellularity in the histological sense would need to be considered perhaps through mechanisms such as micrographic analysis [13], machine learning [3] and/or with further insights garnered from extant projects [36], where cellular type distributions were meticulously considered. Gene expression profiles might also be explored for phenotypic characterization. Model checking has also previously been performed, primarily for molecular species concentrations, but also with respect to their distributions over space and time [63,64,65]. Once convincing simulations are operational at a small scale with verification protocols, the simulations can then be scaled up using HPC. The use of HPC, especially in the domain of GPGPU, is planned for upcoming work.

## Data Availability

The data presented in this study is available in the Supplementary Materials. BENCHMARKING.xlsx contains the raw data gathered during benchmarking. ReactionModels.zip contains the arbitrary CRNs of various topologies generated with SBML-Constructor and subsequently benchmarked with NGSS, along with the multicellular layers. A version of UM3D is publicly available at https://github.com/RichardMatzko/UnrealMulticell3D (accessed on 1 December 2022) via the download of UM3D_Vers_SimplestCell.zip. Other software and code may be made available in the future via the aforementioned GitHub profile.

## Supplementary Materials

The following supporting information can be downloaded at: https://www.mdpi.com/article/10.3390/genes14010154/s1: Video S1: S1 UM3D demo.mp4. Video S2: S2 Tickgate Slow Motion.mp4. Video S3: S3 Server Control Over Phenotype.mp4. Video S4: S4 SMB demo.mp4. Video S5: S5 High-Resolution Constrained Stochastic Chain Extension—Pseudo Off Lattice.avi.
